# Application of Intra-Oral Dental Scanners in the Digital Workflow of Implantology

**DOI:** 10.1371/journal.pone.0043312

**Published:** 2012-08-22

**Authors:** Wicher J. van der Meer, Frank S. Andriessen, Daniel Wismeijer, Yijin Ren

**Affiliations:** 1 Assistant professor, Department of Orthodontics, University Medical Centre Groningen, University of Groningen, Groningen, The Netherlands; 2 Postgraduate, Section of Oral Implantology, Academic Centre for Dentistry Amsterdam, Amsterdam, The Netherlands; 3 Professor and chair, Department of Oral Function and Restorative Dentistry, Head of the Section of Oral Implantology and Prosthodontics, Academic Centre for Dentistry Amsterdam, Amsterdam, The Netherlands; 4 Professor and chair, Department of Orthodontics, University Medical Centre Groningen, University of Groningen, Groningen, The Netherlands; University of Toronto, Canada

## Abstract

Intra-oral scanners will play a central role in digital dentistry in the near future. In this study the accuracy of three intra-oral scanners was compared. Materials and methods: A master model made of stone was fitted with three high precision manufactured PEEK cylinders and scanned with three intra-oral scanners: the CEREC (Sirona), the iTero (Cadent) and the Lava COS (3M). In software the digital files were imported and the distance between the centres of the cylinders and the angulation between the cylinders was assessed. These values were compared to the measurements made on a high accuracy 3D scan of the master model. Results: The distance errors were the smallest and most consistent for the Lava COS. The distance errors for the Cerec were the largest and least consistent. All the angulation errors were small. Conclusions: The Lava COS in combination with a high accuracy scanning protocol resulted in the smallest and most consistent errors of all three scanners tested when considering mean distance errors in full arch impressions both in absolute values and in consistency for both measured distances. For the mean angulation errors, the Lava COS had the smallest errors between cylinders 1–2 and the largest errors between cylinders 1–3, although the absolute difference with the smallest mean value (iTero) was very small (0,0529°). An expected increase in distance and/or angular errors over the length of the arch due to an accumulation of registration errors of the patched 3D surfaces could be observed in this study design, but the effects were statistically not significant.

**Clinical relevance:**

For making impressions of implant cases for digital workflows, the most accurate scanner with the scanning protocol that will ensure the most accurate digital impression should be used. In our study model that was the Lava COS with the high accuracy scanning protocol.

## Introduction

The basis for prosthetic work in dentistry has traditionally been an intra-oral impression that was subsequently poured in dental stone. The stone model forms the basis for the dental lab to manufacture crowns, fixed partial dentures and frames attached to natural teeth. Stone models are also used for producing frameworks for implant cases. This traditional workflow has proven itself in clinical practice, even though impression materials are prone to dimensional changes due to on-going chemical reactions [1] and stone will show expansion due to secondary reactions whilst setting [2]. Aforementioned dimensional changes may very wellresult in a misfit of the cast restorations. The misfit of fixed partial dentures on natural teeth will result in forces on the underlying teeth. Natural teeth however can move 25–100 µm in axial direction and 56–108 µm in lateral direction [3], [4] and adapt to a slightly different position in the bone due to the periodontal ligament should there be a slight misfit of the prosthetic work. Implants on the other hand will only show a range of motion of 3–5 µm in axial direction and 10–50 µm in lateral direction after osseointegration due to compression of the bone [4]. Ill-fitting framework will generate stress on the implants which may have a biological effect on the bone-implant interface [5], [6]. Also prosthetic complications as screw loosening or fracture may be related to ill-fitting framework fit [7]. A finite element analysis (FEA) study has also shown that passive fit will distribute masticatory forces more evenly over the implants [8]. The aforementioned factors have resulted in the paradigm that passive fit of the framework is one of the key factors for long-term success in implant dentistry [9], [10] stressing the importance of a reliable and precise impression procedure. Several strategies have been developed to ascertain passive fit [3], [11]. Even though none of the techniques has proven to be a panacea, the application of industrial-based digital production workflows is a solution that seems to gain popularity. As the impression procedure is at the origin of the workflow, the data collected during this phase is important as errors introduced in this phase will reverberate in the rest of the workflow. An intra-oral scanner could overcome some of the errors associated with traditional impression taking [12] and cast production [13], as digital output data can be fed directly into a digital workflow. The assessment of the accuracy of traditional impression materials has primarily been performed using linear or 3D measurements. The accuracy deviations that were found in those studies have been expressed in µm or percentages [14], [15], [16].

When considering accuracy one is inclined to consider only what we can refer to as “local accuracy” where the scan of a small geometrical form is compared to the original form and the difference between the two forms can be considered as the accuracy of the scanner. This would hold true for accuracy needed for single crown units in dentistry. This accuracy has been determined for intra-oral scanners by several authors [17], [18], [19]. Another form of accuracy would be the accuracy over more units across the dental arch, which could be referred to as “general accuracy”, resembling the accuracy necessary for the production of multi-unit fixed partial denture on natural teeth or implants. This form of accuracy is especially interesting if one considers full arch impressions for implant framework. In those cases the accuracy of the full-arch impression and the distance between the implants leaves less room for errors due to the rigidity of the bone-implant interface [11]. Although the dichotomy between “local” and “general” accuracy may seem immaterial at first, the rationale behind it is that all the intra-oral scanners build their 3D models by combining several 3D images made of the same section of the model but from different angles. The composition of the different 3D patches inevitably leads to registration errors that may vary in magnitude depending on the scanning technology and the registration algorithms used [20] [21], [22]. *Even though other stud*ies have tried to establish the accuracy of some of the intra-oral digitizers [17], [18], [19], no consensus exists on how to assess the accuracy of intra-oral scanners. Some have looked at single teeth [18], several teeth in a row [17] or at quadrants [18]. One study has looked at full arch scans [19]. In order to simplify the comparison, the dataset comparison was always reduced to a single number depicting the difference between the dataset of the scanner and the golden standard. In our study we wanted to consider the accuracy necessary for multi-unit framework on implants and a single number does not indicate possible error fluctuations over a longer span in those cases. We have therefore chosen to measure the distance and angular changes over a longer span between simulated implants generating multiple numbers that can be compared.

The objective of this study was to assess the “general accuracy” of three commercially available intra-oral scanners, that employ different scanning technologies to obtain the 3D images, for the application in the digital workflow in implant prosthetics.

## Materials and Methods

### The model

Three high precision PEEK (polyether ether ketone) cylinders were manufactured by Createch Medical (Createch Medical, Mendaro, Spain) with an accuracy of 2 µm. PEEK was chosen for its excellent mechanical and chemical properties and to avoid a reflective surface that a metal cylinder would provide, as all intra-oral scanners have problems scanning reflective, shiny surfaces.

On a full arch stone model of a volunteer, the teeth 36, 46 and 41 were ground to gingival level. Subsequently a hole was drilled in the stone and implant analogues were placed in the prepared cavities and embedded in stone. The high precision cylinders were then screwed on the implant analogues.

### The intra-oral scanners

**Figure 1:**
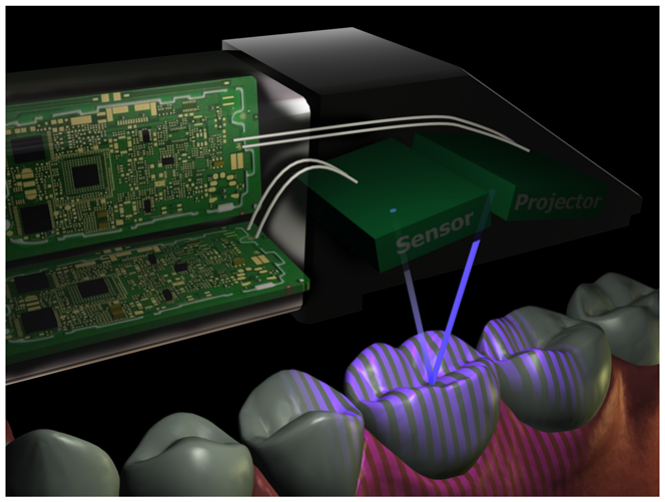
The technical principle of the CEREC scanner. The Cerec projects a light stripe pattern on the object. As each light ray is reflected back on the sensor, the distance between the projected ray and reflected ray is measured. Because the fixed angle between the projector and sensor is known, the distance to the object can be calculated through Pythagoras theorem, as one side and one angle (the fixed angle) of the triangle are now known. Hence the name “triangulation”.

**Figure 2:**
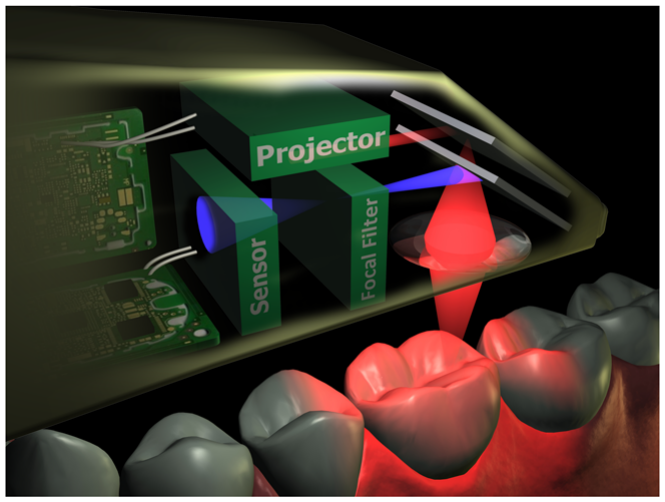
The technical principle of the iTero scanner. The iTero scanner uses confocal laser scanning in which a laser beam (red) is projected on an object. Via a beam splitter, the reflected beam (purple) is led through a focal filter so that only the image that lies in the focal point of the lens can project on the sensor. As the focal distance is known, the distance of the scanned part of the object to the lens is known (the focal distance). To scan the whole object, the lens is moved up and down, each time projecting a part of the object onto the sensor.

**Figure 3:**
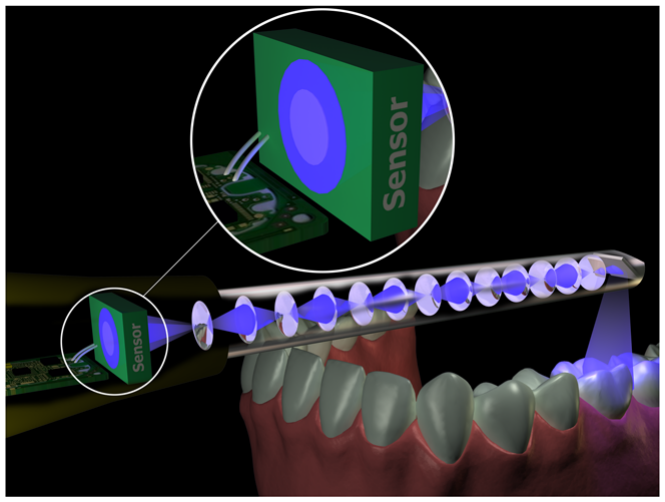
The technical principle of the Lava COS scanner. The Lava COS uses “active wavefront sampling” to calculate the 3D model of the teeth. For this the image reflected from the teeth is led through a lens system and eventually projected onto a sensor. If the image is in focus, the distance of the object coincides with the focal length of the lens. If the image is out of focus, the distance from the lens to the object can be calculated from the size of the blurred image through a simple mathematical formula.

The intra-oral scanners used in the study were the CEREC AC with the CEREC bluecam (Sirona Dental Systems Gmbh, Bensheim, Germany) with software version 3.85, the Cadent iTero (Cadent Inc, Carlstadt, USA) with software version 3.5.0 and the Lava COS (3M Espe, St. Paul, USA) with software version 2.1). All 3D scanners measure the distance from the scanner's sensor-tip to the object with different technologies to convert the optical data to a 3D model. The CEREC AC system employs light stripe projection and active triangulation (Figure 1) to generate 3D images [23]. The Cadent iTero scanner employs a parallel confocal imaging technique [24] for capturing 3D images (Figure 2). The Lava COS uses active wavefront sampling [25] to obtain a 3D model of the dentition (Figure 3). Both the CEREC AC and the Cadent iTero capture single 3D frames that are stitched with other frames to compose a complete 3D model in a short registration cycle. After each cycle the user can proceed to scan the next part of the model. After the scanning procedure the model can be uploaded to respectively CEREC or iTero for post processing. The Lava COS is a 3D video system that captures 20 3D frames per second, which are registered real-time. After the scanning procedure a post processing cycle is necessary to recalculate the registration and compensate for potential errors, resulting in a high resolution model that is uploaded to 3M.

### Dusting or powdering

The iTero scanner does not need special preparation of teeth to be scanned. Before scanning with the Lava COS, teeth need to be dusted with Lava Powder (3M Espe, St. Paul, USA), a titanium-oxide powder. The latter has to do with the technology the scanner employs. The dust particles on the teeth are used for registration of the 3D patches obtained during scanning. When employing the CEREC AC, a matte finishing needs to be applied to the surface to be scanned to prevent reflections. For this purpose the surface is covered with a thin layer of Optispray (Sirona Dental Systems GmbH, Bensheim, Germany). To correctly mimic the clinical situation, the models were prepared according to the manufacturer's instructions with the appropriate powder before scanning the model. To avoid possible errors due to powder contamination, the order of scanning was decided to be

1. the iTero, as it required no powder
2. the Lava COS, as it required only light dusting
3. the CEREC, as it required the complete surface to be covered with a thin layer of Optispray.

### 3D scanning

**Figure 4:**
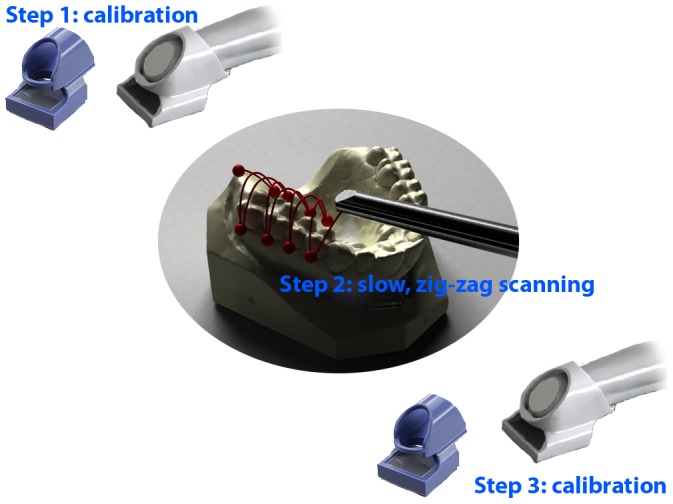
The hi-res scanning protocol for the Lava COS scans. The scanning protocol for the scans for the Lava COS is the normal scanning protocol, except that the scan-path is a slow zigzag scan and that at the end of the scan a second calibration is performed.

The model was attached to a table and scanned 10 times with three different intra-oral scanners: the iTero (Cadent Inc, Carlstadt, USA), the Lava COS (3M Espe, St. Paul, USA) and the CEREC (Sirona Dental Systems GmbH, Bensheim, Germany). The manufacturers were asked for the protocol for high accuracy scanning as would be used for scanning implant locators and for special considerations for this type of scanning, e.g. calibration of the scanning unit or modification of the scanning protocol. The iTero and the CEREC had only one scanning protocol for all cases and did not distinguish between normal scanning and high accuracy scanning. The Lava COS had a high accuracy scanning protocol and subsequent calibration protocol. The normal Lava scanning protocol consists of a calibration with small calibration block before the intra-oral scan starts followed by scanning of the teeth according to a non-prescribed scan path. The high accuracy scanning protocol for scanning implant abutments consists of a calibration with the aforementioned calibration block followed by a slow zig-zag scanning of the dentition. After the scan the calibration with the calibration block is performed for a second time (Figure 4). The calibration measurements are used to calculate and compensate for errors that have occurred during scanning.

All the scans were performed according to the instructions of the manufacturer by a dentist proficient with the specific intra-oral scanner. As only the iTero scanner does not require dusting or powdering of the model, the iTero scanner was used first to scan the model 10 times with a 10 minute interval between the scans. After this the model was dusted according to the instructions for the Lava COS with Lava Powder (3M Espe, St. Paul, USA) and the model was scanned 10 times with this scanner with a 10 minute interval between the scans. After the model was cleaned with a soft brush, the model was sprayed with Optispray (Sirona Dental Systems GmbH, Bensheim, Germany) according to the instructions of the manufacturer and 10 consecutive scans were performed with a 10 minute interval. All the scans of the different scanners were uploaded to the respective companies and returned after post-processing.

The physical model was cleaned with a soft brush and sent to Createch Medical (Mendaro, Spain) where it was scanned under strictly controlled conditions (temperature, humidity and vibrations) with a ultra-precision contact scanner with a precision of 0.1 µm (Leitz PMM 12106). The latter digital model formed the reference data set.

### 3D measurements

**Figure 5:**
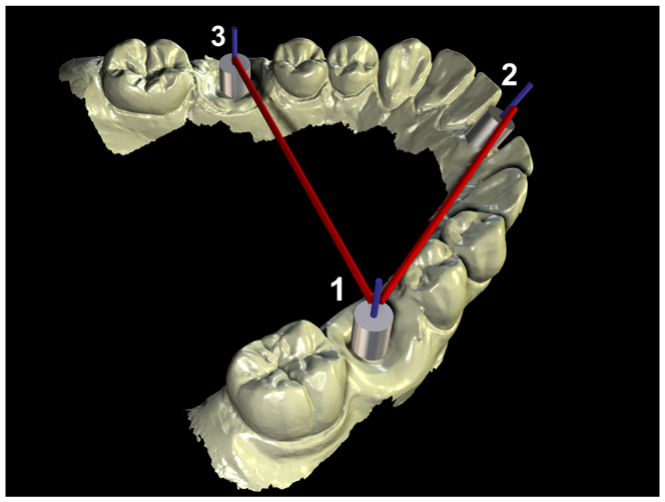
The measurements were made between the centers of the high-precision cylinders. Three 3D CAD models of the cylinders in the model were imported and registered with each of the scanned equivalents. The distance between the centre-lines was measured in the software using a linear measurement tool. The angular deflection of the cylinders was measured with an angular measurement tool, using the cylinder at the location of the lower right molar as the baseline.

The distance and the angle between the centres of the high precision cylinders were used to assess the accuracy of the different scanners (Figure 5). For this each of the scans was imported in industrial reverse engineering software Rapidform (Rapidform, INUS Technology Inc, Seoul, Korea), where each of the cylinders was isolated as a separate object. Three 3D CAD models of the cylinders were subsequently imported and registered with each of the scanned equivalents. This was done to enable the proper construction of the centre-line of each cylinder.

To validate the precision of the registration algorithm a CAD cylinder, like the one used in the study, was imported in the Rapidform software. There it was duplicated and the second cylinder was subsequently moved to another location in the 3D space. The two cylinders were then registered and the difference between surfaces of the two cylinders was calculated by the software. As the cylinders are perfectly identical, the surfaces of the cylinders should ideally match perfectly. The experiment was repeated ten times and the mean of the registration error was calculated. The mean error of the registration procedure was 1.4 nm (+/−0.9 nm).

The distance between the centre-lines was measured in the software using a linear measurement tool. The angular deflection of the cylinders was measured with an angular measurement tool, using the cylinder at the location of the lower right molar as the baseline. The measurements were not broken down in x-, y- and z-components as the objects coordinate system could not properly be matched with a world coordinate system. As there is no true common coordinate system, the different models could only be registered in a virtual common coordinate system. As the registration is based on the surface of the models and as these will show minor errors, the positions of the models will differ slightly. This will introduce an error in their relative positions and makes it unreliable to compare measurements broken down in x-, y- and z-components. The measurements were noted in a table and compared to the same measurements made on the reference data set. A one-way ANOVA was performed to compare the differences between the 3 systems (P<0.05).

## Results

**Figure 6:**
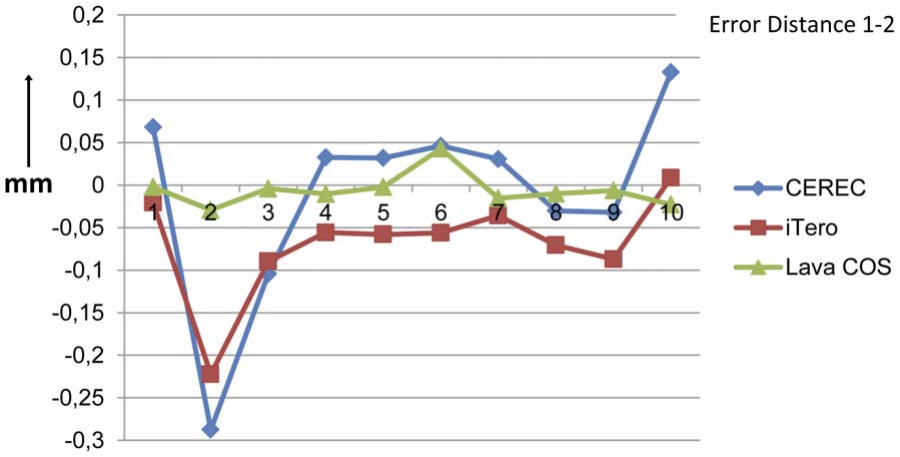
The distance errors between the cylinders 1 and 2 in millimeters for the three intra-oral scanners. The smallest distance error between cylinders 1 and 2 was −22,0 µm (Lava COS), while the largest error was −287,5 µm (CEREC). The Lava COS scanner showed the smallest mean distance error and also showed the smallest variations.

**Figure 7:**
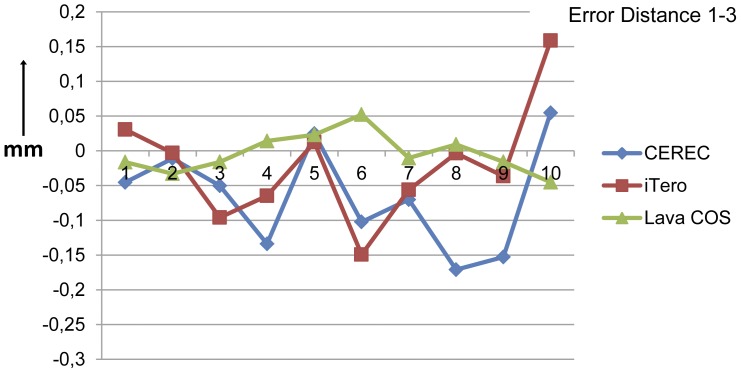
The distance errors between the cylinders 1 and 3 in millimeters for the three intra-oral scanners. The smallest distance error between cylinders 1 and 3 was −32,0 µm (iTero), while the largest error was −171,1 µm (CEREC). The Lava COS scanner showed the smallest mean distance error and also showed the smallest variations.

The results are summarized in Table 1 and Table 2. The absolute distance errors ranged from 2,2 µm (Lava COS) to 287,5 µm (CEREC) (Figures 6 and 7). The mean of the distance errors of both the measured distances of the Lava COS, respectively 14,6 µm (95% confidence interval: 6,7 µm–22,4 µm) for the distance 1–2 and 23,5 µm (95% confidence interval: 14,7 µm–32,3 µm) for the distance 1–3. These values were the smallest compared to the CEREC and the iTero scanner. The confidence interval for the Lava COS was the smallest demonstrating that the variations were the smallest. The distance errors of the CEREC were the largest, respectively 79,6 µm (95% confidence interval: 31,8 µm–127,4 µm) for the 1–2 distance and 81,6 µm (95% confidence interval: 49,1 µm–114,2 µm) for the 1–3 distance. All of the scanners had errors both in the positive and the negative range.

**Table 1:** Absolute errors in the distance between the cylinders in micrometers.

	CEREC		iTero		Lava COS	
	ABS Error 1–2	ABS Error 1–3	ABS Error 1–2	ABS Error 1–3	ABS Error 1–2	ABS Error 1–3
**MEAN**	79,6	81,6	70,5	61,1	14,6	23,5
**SD**	77,1	52,5	56,3	53,9	12,7	14,2
**CI** (95%)	31,8–127,4	49,1–114,2	35,5–105,4	27,7–94,5	6,7–22,4	14,7–32,3

**Table 2:** Absolute errors in the angle between the cylinders in degrees.

	CEREC		iTero		Lava COS	
	ABS Error 1–2	ABS Error 1–3	ABS Error 1–2	ABS Error 1–3	ABS Error 1–2	ABS Error 1–3
**MEAN**	0,6303	0,4378	0,3451	0,4192	0,2049	0,4722
**SD**	0,5499	0,3211	0,3382	0,1667	0,0440	0,1436
**CI** (95%)	0,2894–0,9711	0,2388–0,6367	0,1355–0,5547	0,3159–0,5226	0,1776–0,2322	0,3831–0,5612

**Figure 8:**
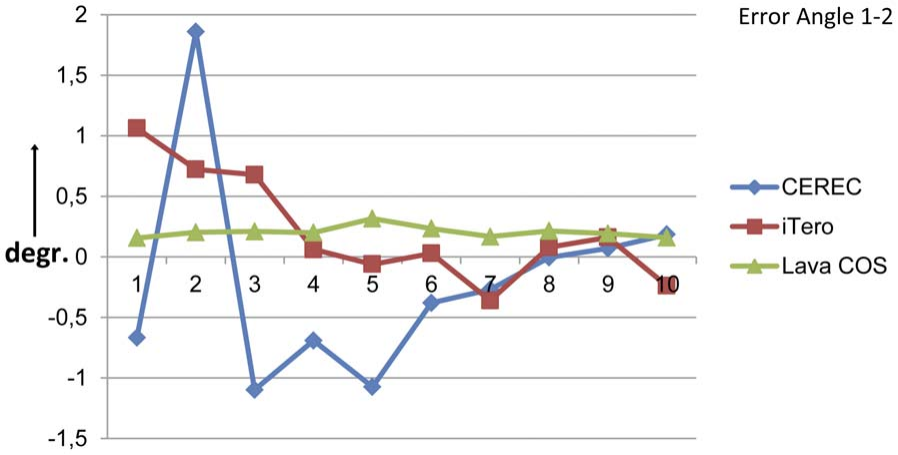
The angulation errors between the cylinders 1 and 2 in degrees for the three intra-oral scanners. The angulation errors were small and ranged from −0,0061° (CEREC) to 1,8585° (CEREC). The Lava COS showed the smallest mean angulation error and also the smallest variations. The Lava COS also showed only positive errors.

**Figure 9:**
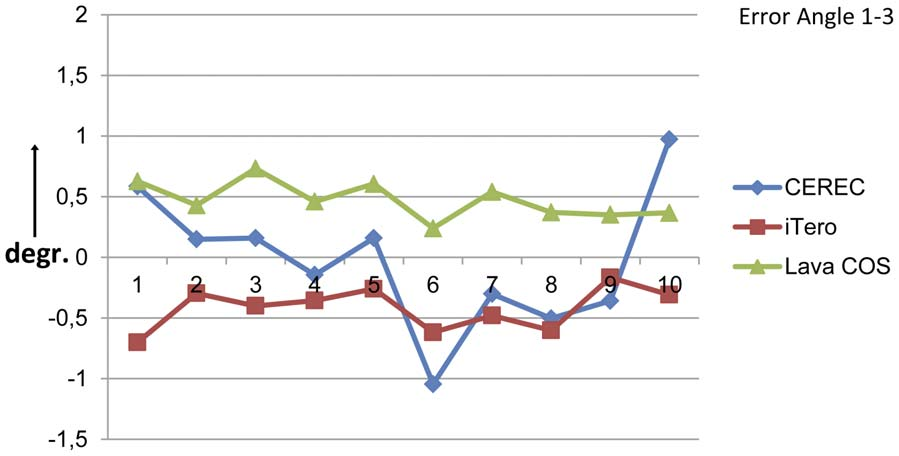
The angulation errors between the cylinders 1 and 3 in degrees for the three intra-oral scanners. The angulation errors were small and ranged from −0,1447° (CEREC) to 1,0456° (CEREC). The iTero showed the smallest mean angulation error. The Lava COS showed the smallest variations. The Lava COS showed only positive errors, while the iTero showed only negative errors. Only the Lava COS showed consistent positive errors in all cases, this could be regarded as an offset which may be compensated.

The angulation errors are shown in the figures 8 and 9. The mean of the absolute angulation errors ranged from 0,0061° (CEREC) to 1,8585° (CEREC). The mean absolute angulation errors for the cylinders 1–2 was the smallest for the Lava COS: 0,2049° (95% confidence interval: 0,1776°–0,2322°) and the largest for the CEREC: 0,6303° (95% confidence interval: 0,2894°–0,9711°). For the cylinders 1–3 the smallest mean absolute angulation error was provided by the iTero : 0,4192° (95% confidence interval: 0,3159°–0,5226°) and the largest by the Lava COS: 0,4722° (95% confidence interval: 0,3831°–0,5612°). The confidence interval for both the angulation errors 1–2 and 1–3 was the smallest for the Lava COS, indicating that the Lava COS had the smallest variations in its angulation errors. The CEREC had angulation errors in both the positive and negative range between the cylinders 1–2 and 1–3. The iTero showed a similar distribution for the angulation errors 1–2, but showed only negative values for the 1–3 measurements. The Lava COS was the only scanner that showed errors in the positive range for all measurements.

No statistical difference was found between the three groups.

## Discussion

To our knowledge, this is the first study that compares three different intra-oral scanning technologies. The present study analysed the accuracy of three intra-oral scanners by determining the distance and angulation errors in vitro. The results show that the Lava COS has the smallest mean distance errors and the least variations in the measurements. In the angulation errors, the Lava COS showed the smallest mean error between cylinder 1–2 and the CEREC the largest mean error. The difference between the smallest and largest error was very small (0,4254°). Between cylinders 1–3 the iTero showed the smallest mean error and the Lava COS the largest mean error. The difference between the smallest and the largest error was even smaller: 0,053°. The Lava COS had the smallest confidence interval in angular and linear measurements, indicating that this scanner has the lowest variation in its measurements. The Lava COS was also consistent in the angular errors as their range was small and all the values were positive. Only one other study has compared different intra-oral scanners. Ender and Mehl [19] have compared the Lava COS and the Cerec to determine which scanner is more accurate compared to the cast of an Impregum impression. In their study, the accuracy was defined by the terms “trueness”: the deviation of the model with respect to the true size of the object, and “precision”: the fluctuation of the different measurements. The “trueness” of the Lava COS was better than that of the CEREC and both were better than an Impregum impression. The “precision” of the CEREC was better than the Lava COS which was comparable to the Impregum impression. The high accuracy scanning protocol was not used in that study. Special software was used to superimpose datasets and the difference between the two models based on measuring points was calculated. This resulted in one value for the accuracy of the scanner.

The various scanners used in our study use different technologies to determine the spatial coordinates of the scanned object. Differences found between the three scanners may be related to measurement errors inherent to the technology employed. To improve the resolution of the 3D scan, CEREC has switched from white to blue light which has a shorter wavelength leading to a higher accuracy [18]. Apart from the differences in the technology of data acquisition, the CEREC and iTero scanners are point-and-click systems, while the Lava COS is a video system. This may explain both the similarities between the CEREC and iTero measurements and the differences with the results of the Lava COS. In the point-and-click systems, the 3D surfaces should be scanned with at least a one-third overlap of the adjoining surface. The registration of the neighbouring surfaces will occur on the basis of this overlap. In the video system with a frame rate of 20 images per second, the overlap of the images will most likely be larger than the aforementioned one-third which could lead to a better surface registration. Differences in the results may also occur due to the registration of the 3D images and in the rest of the post-processing procedure. The Lava COS uses powder particles as markers as an extra toolfor the computer to join the different pieces of the 3D model. How the registration takes place and what algorithms are used in the different scanners is not shared knowledge. But algorithms that involve registration based on surface overlap are most likely. As registration errors, however minute, will always occur in registration procedures [26], one expects an additive effect of these errors over the length of the arch. When comparing intra-oral scanners in full arch impression procedures, it would be interesting to involve the influence of the length of the span to assess the expected additive effect of the registration errors that may occur. The aforementioned effect could be observed in our experiments for the CEREC and the Lava COS when considering the distance accuracy and for the iTero and the Lava COS when considering the angular accuracy. The differences however were very small and statistically not significant. Mehl et al found a decreasing accuracy when comparing single tooth images to quadrant images for the CEREC Bluecam intra-oral scanner [18] which could be explained by an accumulation of registration errors. In the study of Ender and Mehl [19] an increase in the deviations between the models in certain areas were noted, but these can be explained by the registration procedure. The algorithm most likely tried to register the surfaces in such a way that the overall mean deviation between the surfaces is the smallest and this may conceal an increase in deviations between the surfaces and makes interpretation of deviations difficult. A best fit algorithm on basis only of the area where the scanning was started may have shown a possible increase in deviations in their study.

In the study of Ender and Mehl [19] a mean “trueness” of 49±14.2 µm was found for the CEREC and 40.3±14.1 µm for the Lava COS. The difference from our data is most likely resulting from a different research model in their study and a high accuracy scanning protocol for the Lava COS in our study. In their study a 3D comparison was made between the models, where the computer calculates the difference between the surface points of the models. These measurements are usually expressed in a mean value for the error between the surfaces. In our study the linear and angular measurements were made as the accuracy of the distance and the angulation between implants can show the error that will be introduced at the inlet of a digital workflow. Other methods, like the aforementioned 3D comparison of digital models, will also generate a number that will generally reflect the accuracy of a model. However this number will not express the exact error between implant positions nor will it show errors in angulation that may occur or a possible increase in the distance and angular errors over distance.

In future studies other video-scanners should be involved as we have compared two point-and-click systems (CEREC and iTero) with one video system (Lava COS) in the present study. Differences in outcome could be explained with differences between the technologies as explained above. The amount of cylinders on the model should be increase to gain a better insight in possible increase in deviations over the length of the span. The number scans may be increased to increase the reliability of the study. Also a comparison with a traditional impression material, like Impregum, should be added to enable a comparison with the traditional workflow. A comparison between the normal scanning protocol and the high accuracy scanning protocol should also be included.

### Conclusions

1. The Lava COS in combination with a high accuracy scanning protocol resulted in the smallest and most consistent errors of all three scanners tested when considering mean distance errors in full arch impressions for both measured distances.
2. For the mean angulation errors of the three scanners tested, the Lava COS had smallest errors between cylinder 1–2 and the largest errors between cylinder 1–3, although the absolute difference with the best mean value (iTero) was very small (0,0529°).
3. In the Lava COS the angulation errors were very consistent with a small confidence interval value.
4. An expected increase in distance and/or angular errors over the length of the arch due to an accumulation of registration errors of the patched 3D surfaces could be observed in this study design, but the effects were statistically not significant.

### Clinical relevance

For making impressions of implant cases for digital workflows, the most accurate scanner with the scanning protocol that will ensure the most accurate digital impression should be used. In our study model that was the Lava COS with the high accuracy scanning protocol.
